# CAREUP: An Integrated Care Platform with Intrinsic Capacity Monitoring and Prediction Capabilities

**DOI:** 10.3390/s25030916

**Published:** 2025-02-03

**Authors:** Marcin Kolakowski, Andrea Lupica, Seif Ben Bader, Vitomir Djaja-Josko, Jerzy Kolakowski, Jacek Cichocki, Jaouhar Ayadi, Luca Gilardi, Angelo Consoli, Irina Georgiana Mocanu, Oana Cramariuc, Lionello Ferrazzini, Eva Reithner, Magdalena Velciu, Barbara Borgogni, Sofia Rivaira, Sara Leonzi, Giacomo Cucchieri, Vera Stara

**Affiliations:** 1 Institute of Radioelectronics and Multimedia Technology, Warsaw University of Technology, 00-661 Warsaw, Poland; 2 ECLEXYS Sagl, 6826 Riva San Vitale, Switzerland; 3 OCTILIUM Sagl, 6830 Chiasso, Switzerland; 4 Centrul IT Pentru Stiinta si Tehnologie (CITST), 020771 Bucharest, Romania; 5 Computer Science Department, Faculty of Automatic Control and Computers, National University of Science and Technology Politehnica Bucharest, 060042 Bucharest, Romania; 6 EURAG Österreich, 1170 Wien, Austria; 7 Ana Aslan International Foundation, 020771 Bucharest, Romania; 8 Medea S.r.l., 50144 Florence, Italy; 9 IRCCS INRCA, National Institute of Health and Science on Aging, 60124 Ancona, Italy

**Keywords:** older adult support, intrinsic capacity, machine learning, ICT platforms

## Abstract

This paper describes CAREUP, a novel older adult healthy aging support platform based on Intrinsic Capacity (IC) monitoring. Besides standard functionalities like storing health measurement data or providing users with personalized recommendations, the platform includes novel intrinsic capacity assessment and prediction algorithms. Older adults’ performance is continuously monitored in all five IC domains—locomotion, psychology, cognition, vitality, and sensory capacity—based on measurement results and answers to questionnaires gathered using the platform’s mobile applications. The users are also presented with a machine learning-based prediction of how their intrinsic capacity might change over the following years. The platform’s operation was successfully tested with the participation of older adults and their caregivers in three countries: Austria, Italy, and Romania.

## 1. Introduction

According to the World Health Organization (WHO) [1], by 2050, almost 22% of the world’s population will be aged 60 years or older. This percentage might be even higher in Western Europe due to the currently low birth rates. In this rapidly aging society, it will be of prime importance to support older adults in maintaining their health and prolonging their independent day-to-day functioning. The rapid advancement of information and communications technologies (ICT), including personal electronics, opens up an opportunity to use modern developments to enhance elderly care. Over several years, there many ICT care platforms have been developed, mainly aiming to monitor elderly users’ health parameters [2] and provide alerts in the case of dangerous events, e.g., falls or low sugar levels [3]. The above functions reduce caregivers’ burden, which brings about multiple social and economic benefits [4].

In this paper, we describe an integrated care platform developed within the CAREUP (An Integrated Care Platform Based on the Monitoring of Older Individual Intrinsic Capacity for Inclusive Health) Active and Assisted Living (AAL) project [5]. The CAREUP platform distinguishes itself from other available solutions by offering continuous assessment and prediction of elderly users’ intrinsic capacity (IC). Intrinsic capacity is a novel concept introduced by the WHO that aims to assess older adults’ state based on their individual capacities in physical and mental domains rather than the typically used health parameters and prevalent comorbidities.

Intrinsic capacity is assessed in five domains: locomotion, psychological, vitality, cognition, and sensory capacity. The CAREUP platform performs user assessments in each of them. Additionally, it predicts how users’ performance may change in the following years so that they can take preventive actions suggested by the platform in the form of a personalized care plan.

The rest of the paper is structured as follows. In Section 2, we reference other care platforms described in the literature. Section 3, Section 4 and Section 5 contain descriptions of the platform concept, the adopted design principles, and the technical implementation of the platform. The platform’s main novelty (IC evaluation and prediction) is described in Section 6. The results of the pilot tests are presented in Section 7. Section 8 concludes the paper.

## 2. Healthy Aging Support Systems

Over the last decade, several care platforms intended for use by elderly adults have been designed and implemented. The main goal of all such platforms is to improve users’ quality of life and mitigate or delay adverse health outcomes due to aging. The extent to which these goals can be achieved depends on several platform traits, like the platform’s architecture and functionalities.

### 2.1. Architectures

Elderly support platforms implement various architectures. The simplest one comprises a single sensor and a dedicated server processing measurement results. Such platforms are usually used to supply users with services of a limited scope and target one health domain, e.g., physical capacity. Examples include platforms processing data from FitBit sensors [6] (ECG, acceleration, and GPS location) or custom sensors like an activity sensor in [7]. Instead of dedicated sensors, smartphone applications can sometimes be used [8].

More advanced platforms aggregate results from multiple sensor types and applications. Such solutions adopt one of two approaches; they either use independent infrastructure or are a middleware platform integrating data from sensor vendors’ platforms.

In the first approach, the platforms have their own applications for data gathering and download data from sensors [9]. Such an approach allows all of the elements of the solution to be tailored to the elderly person’s needs. This is especially important if the platform uses custom sensors or implements novel algorithms requiring raw measurement data, which the applications of the device manufacturers might not supply. Keeping the data inside one platform might also positively affect privacy and security [10]. The downside of this architecture is the need to implement a direct connection between the sensors and the platform, which may not be possible due to APIs being closed, which can result in the platform’s incompatibility with some off-the-shelf devices.

The second approach is to implement a platform to act as a middleware service aggregating data from other platforms and services [6,11,12]. In this solution, sensors send data to the respective vendors’ platforms, and the middleware platform then accesses the data through a secure protocol, e.g., OAuth [6]. The platform usually implements only the data processing algorithms and user interfaces. It may integrate data from several different vendors [12] or spaces, e.g., home platforms and hospitals, as in [11]. This approach allows for some flexibility when choosing the devices to be used by the user. If the vendor supplies an API to access data stored in their cloud services, using a particular device should not be a problem. However, as API accessibility may change, it might not always be the case.

Given the potential complexity of both approaches, some designers have decided to implement platforms using cloud services offered by a single company. For example, in [13], the authors presented a platform implemented using Google products, where devices send data to Google Cloud, which is integrated with the Google Home System and Google Assistant for communication with the user.

### 2.2. Functionalities

The suite of offered functionalities is the critical factor determining the platform’s success. The most common functionality is monitoring of the user’s health using several sensors or questionnaires [14] and storing the recorded data. The data may be either automatically processed to profile the user and provide her/him with personalized recommendations or be presented to the user’s caregivers or medical professionals [9]. The platforms may also include a video-chat option, allowing users to contact doctors and get counsel without going to a medical facility [15].

The platforms can also act as activity monitors [16], which track the user’s daily activities to detect deviations that may occur due to a reduction in the user’s capabilities or illness. Activities are sometimes paired with location data to provide additional context [17]. Information on the user’s location can also be used to supply caregivers with geofencing services [18] or for simple navigation [19].

The other essential function of many platforms is patient coaching and education. Coaching is performed in several aspects. The most popular is physical fitness, where the user is presented with personalized exercise programs [20]. However, some platforms also advise users on healthy lifestyle, nutrition, and their psychological and cognitive spheres [14].

Besides educating the user, coaching may also benefit informal caregivers. For example, the platform presented in [21] has a coaching module dedicated to caregivers. Additionally, it includes a virtual caregiver module that provides the user with advice when a real-life caregiver is not present. Such platforms may also reduce caregivers’ burden by controlling drug delivery [22] so that they do not have to be present at all times to administer drugs themselves.

Recent advancements in AI, especially in the domain of large language models, have enabled implementing more sophisticated coaching services using AI chatbots. The authors of [19] proposed the extension of the platform’s capabilities with an interactive ChatGPT-based assistant to answer user questions. Although such an approach is interesting, it poses some dangers, as the chatbot’s answers might be misleading and might put the user’s health in danger, e.g., by suggesting incorrect medicine dosages.

Generally, studies performed to date show that adhering to the proposed guidelines leads to visible improvement [14]. However, the sole presentation of the training plan alongside possible gains does not guarantee active user participation [7]. Therefore, a more complex approach is needed.

One way to motivate users to use the platform actively is to introduce a social networking component [19,23]. Active participation may also require the platform to be accessible and intuitive for use use by elderly users [7], which requires the implementation of dedicated interfaces

### 2.3. User Interfaces

The most popular user interface is either a smartphone [24] or a tablet [9]. Mobile applications usually enable the input and display of data using simple interfaces designed with older adults in mind (large fonts and buttons).

The other typical interface implementation is via web applications [9,24]. These applications are usually intended for medical professionals and caregivers, as they include detailed graphs and information about the user’s health state. However, some solutions exist where a web browser is used as a primary means to communicate with the user [23].

The above interfaces offer many capabilities. However, not all seniors may use them fluently. Therefore, it may be a good idea to explore other possibilities. In [25], the authors proposed a TV-based interface for the platform. Using a TV is a good idea, as it is the most prevalent device in the homes of the elderly, and using TVs reduces the learning curve needed to start using the platform.

Some platforms may introduce non-standard ways of communicating with the user. For example, the platformed proposed in [23] uses ambient displays to communicate a new message received in a social network (a curtain is illuminated and moved with a fan or a bulb changes color). The proposed solution also enables users to tap a tangible device to alert emergency contacts.

Interaction between the user and the platform may also be implemented using robots [26]. Robots can be used to communicate with users and help them in daily tasks like carrying objects [27]. Although such solutions may be useful, their price significantly limits their applicability.

Some caregiver-oriented platforms may not require patient interaction at all. For example, cameras can be used for activity and emotion recognition, as in [28]. Although such systems may supply algorithms with interesting data that are difficult to obtain in other ways, they might be treated as an invasion of privacy.

### 2.4. Assessment and Predictive Capabilities

Giving users personalized recommendations and providing them with health-state estimates requires assessment of their health state and capabilities. Such assessment is typically performed based on gathered data. Health state can be easily assessed based on medical device measurements, e.g., blood pressure measurements to diagnose high blood pressure [29]. Physical capacity can scored based on the user’s activity [12] or data from dedicated sensors, enabling the performance of standardized tests like the Short Physical Performance Battery (SPPB) [20]. The psychological aspects of a user’s life are usually assessed based on standardized questionnaires [9,14].

Besides assessment, such platforms may use predictive modules to provide users with information on possible risks and guide them to prevent, or at least delay, the occurrence of illness [30]. Several machine learning-based models for the prediction of elderly users’ health states over a longer time horizon have been proposed in the literature. Such models target all domains, from cognitive functions [31,32] and common comorbidities like diabetes [33] and balance problems [34] to specialized models aimed at specific diseases like renal failure [35].

Given that most studies of predictive health models are relatively recent, few platforms offer such functionalities. Examples of platforms where such risk assessment is performed include those presented in [3,24], which introduced prediction methods for short-term glucose dynamics and frailty risk, respectively.

## 3. CAREUP Platform Concept and Functionalities

**Figure 1:**
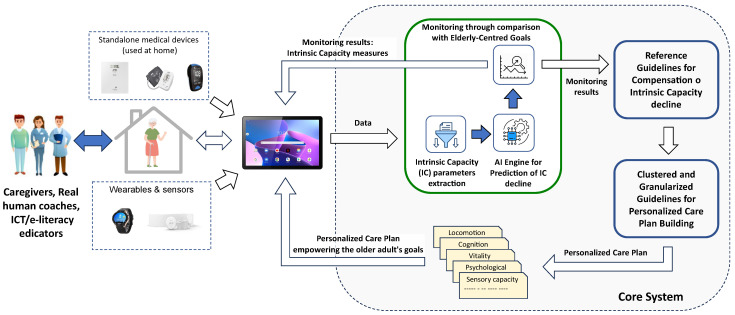
The CAREUP platform concept.

The CAREUP platform is an ICT-based monitoring solution aiming to mitigate the decline of older individuals’ intrinsic capacity. The idea of the platform’s operation is shown in Figure 1. The tablet installed at the user’s home performs two primary roles: it provides access to the platform services (e.g., reviewing monitoring results, playing games offered by the platform, and answering questionnaires) and is a gateway transferring results from wearables, medical devices, and sensors used by the person to the core system.

Processing of the collected data consists of extracting IC parameters describing user performance in particular IC domains, predicting IC degradation, and comparing the results with goals defined by the user. IC evaluation results and goals are the basis for the development of a personalized care plan. The care planning process is focused on the needs, values, and preferences of older individuals. The “Reference Guidelines for Compensation of Intrinsic Capacity decline” represent the gold-standard guidelines established by authoritative bodies, e.g., the World Health Organization, to compensate for the degradation in the intrinsic capacity that is programmed into a computer-interpretable version. The “Clustered and Granularized Guidelines for Personalized Care Plan building” were established by a working group of experts at the start of the project to cluster and granularize the “Reference Guidelines” into more refined guidelines according to a cluster of persons that share the same phenotypes for the degradation of their intrinsic capacities. They issue individualized guidelines and recommendations according to the empowered central goals of individuals. They allow the platform to build a personalized care plan to be communicated to the primary user for implementation.

The plan defines activities to be performed and specifies tools to compensate for identified IC declines in particular domains. Caregivers and coaches have access to monitoring data and the plan; their role is to monitor progress and support plan implementation. IC evaluation is carried out periodically at weekly intervals based on the current sets of collected data. The older adult has to redefine his centered goals periodically (e.g., every six months) or when the IC parameters deteriorate.

## 4. CAREUP Platform Co-Design Methodology

The whole process of the CAREUP design follows the User-Centered Design (UCD) approach as defined by ISO 9241-210:2019 Ergonomics of human-system interaction—Part 210: Human-centered design for interactive systems [36]. The approach is divided into four fundamental activities:

1. User groups are specified, and the context of use is described;
2. A set of specific requirements is defined to create a degree of fit between the device and the user;
3. The design prototypes are produced based on these specifications and are presented to the user in the form of user testing;
4. Once feedback has been received, the last phase is to evaluate the system.

The process is repeated until all user requirements are met. The whole user-driven approach was performed in three countries at the headquarters of end-user partner organizations: EURAG Austria in Austria, INRCA (National Institute of Health and Science on Aging—IRCCS) in Italy, and AAIF (Ana Aslan International Foundation) in Romania.

The first phase of the CAREUP platform definition is analysis of the needs of the end user [37]. Based on the UCD approach, two questionnaires containing closed and open-ended questions were prepared and used to collect data from 30 older adults and 32 informal caregivers. The questions concerned the context in which the CAREUP platform would be used and the IC framework’s ability to clearly convey data on the user’s health.

The results show that most seniors in each country consider the proposed technology necessary. According to the users, the most valued benefits of the platform are being notified of the recording of outlier health parameters, preventing negative outcomes of falling down, real-time monitoring of health values, and evaluating and improving cognitive functions. Besides the perceived benefits, the other factor influencing the willingness to use the CAREUP platform is its ease of use.

User research is vital in comprehending users’ lifestyles, motivations, and challenges. The incorporation of user feedback, prototyping, and testing are crucial steps in understanding users’ needs and expectations and shaping the product accordingly. This user-driven approach prioritizes users’ needs and desires throughout the design process. By understanding user behavior, preferences, and goals, developers can create intuitive, efficient, inclusive, and accessible experiences for all. Moreover, monitoring whether the users feel that the system respects their needs is essential for building trust and maintaining a positive user experience during this process. For that reason, usability, user experience, acceptance, privacy, intrusiveness, and stigma became the primary variables to measure during pre-pilot and pilot testing after the different stages of development.

The users were consulted about the early version of the platform during the pre-pilot stage. The pre-pilot tests assessed and validated the system components to find and solve major usability weaknesses by enrolling 15 older adults and 15 informal caregivers. The pre-pilot tests focused on users’ preferences and usability recommendations to evaluate each component’s acceptability and usefulness/efficiency. In that way, conclusions and users’ proposals were transferred to the technical partners to release an advanced version of the system to be evaluated in the final pilot. The outcomes of the final pilot study are presented in Section 7.

The potential users were also asked about the costs associated with the platform’s use. The expenses that the users would incur include the cost of the devices and a monthly fee to use the platform. The cost of the starting set of devices needed by the platform (tablet and health sensors) was set to EUR 325. If the user already owns some pieces required by the platform e.g., a tablet, a blood pressure meter, etc., this cost would be lowered. The monthly access fee for the platform was set at EUR 7.5, which was accepted as a reasonable amount by the users and caregivers.

Direct comparison of platform pricing might be problematic due to differences in functionalities offered by various platforms. However, even when compared to other elderly care solutions, the price of CAREUP is favorable. For example, the basic functionalities of an another platform for medical data processing and aggregation, MyChart [38], which is used in the USA, are usually included in the user’s health insurance. However, some of the services are additionally paid. One-time consultation concerning the recorded results may cost between USD 7 and 50 [39], which is close to the yearly cost of using the CAREUP platform.

The pricing of other, more limited platforms is also relatively high. A good example is geofencing solutions for older users who have dementia. Single-purpose solutions, e.g., Protection Pendant [40], that send direct alerts to the caregiver without offering any platform benefits start as low as a one-time charge of USD 99 per sensor. More advanced solutions are usually more expensive. For example, the cost of using the BoundaryCare [41] application on a smartwatch owned by the user ranges from USD 25 to 35 per month, depending on the commitment length. The platform allows users to expand the list of monitored parameters, with a compliant oxygen saturation monitor priced at USD 200.

Similar costs are incurred by the users of medical alert systems sending notifications in the case of emergencies such as falling down. The monthly cost of such platforms starts at USD 25 [42].

Given the costs of the other solutions and the rising costs of medical consultations, the CAREUP platform seems to be a cost-efficient solution, the adoption of which could reduce medical expenses incurred both by the user and medical systems.

## 5. CAREUP Platform Implementation

### 5.1. System Architecture

**Figure 2:**
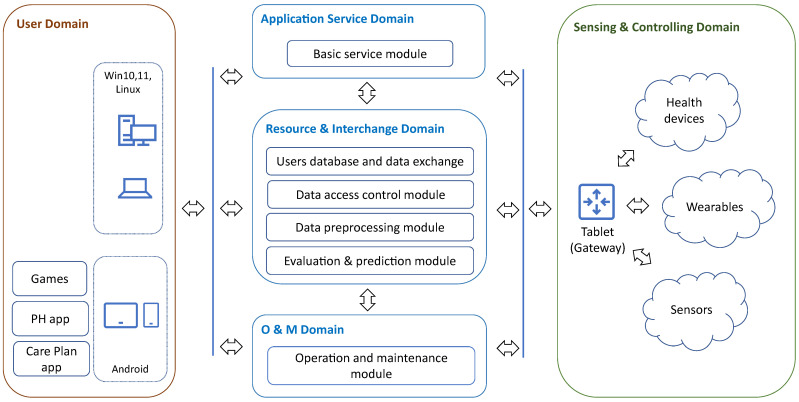
The CAREUP platform architecture (system view).

The platform architecture (Figure 2) follows the ISO/IEC CD 30141 standard [43]. The view presents the main physical system components (e.g., sub-systems, devices, networks, and software modules) of the platform, their distribution, and the topology of their interconnectivity. The devices are grouped into five domains. The user and sensing and controlling domains include devices installed at the user’s home and worn by the user. The other domains comprise software modules running on the core server located in the cloud environment.

### 5.2. User Domain

The CAREUP platform offers two categories of services: local services and RESTFul-based web services, which can be accessed with PCs, notebooks, tablets, and smartphones through embedded Internet browsers. Local services are implemented in Android-based devices (i.e., tablets and smartphones). Although local services are based on autonomous applications, the data collected during their use are sent to the resource and interchange domain.

The platform includes three tablet applications: Care Plan, the Positive Health (PH) application, and cognitive games. Care Plan is the main application with which the end user interacts. Its primary function is to present the user with individualized care-plan tasks, enabling him to reach his healthcare goals. It is also an interface for manually uploading the data from the medical sensors and accessing the PH and cognitive games applications.

The main purpose of the PH application is to collect data on the user’s current self-rated health; mood; and other aspects of his life, such as social participation. The application also implements basic physical tests. The cognitive games application offers the user several games testing her/his logical thinking and reflexes. The game scores are sent to the cloud for eventual evaluation.

### 5.3. Sensing and Controlling Domain

The sensing and controlling domain comprises devices collecting measurement results reflecting the user’s health status. The gateway is a core component of the sensing and controlling domain. In the CAREUP platform, this role is fulfilled by a tablet equipped with interfaces and software that allow for the exchange of data with other domains (using cellular/Ethernet links), in addition to collecting monitoring results (over BLE links) from sensors, wearables, and medical devices used by older individuals at home and during their outdoor activities. The tablet is also used to access care plans, games, mobile apps, computerized questionnaires, and monitoring results.

### 5.4. Application Service Domain

In the current platform implementation, the domain includes a module providing basic platform services: portal access, data presentation, and care-plan preparation. This module comprises a web portal application, a data visualization module, and a care-plan composition module. All run in Docker containers on the core server.

To access most CAREUP services, the user must log into the CAREUP portal with a browser. The service portfolio and user interface depend on the user category. Primary users obtain access to the care plan and data presenting their performance in IC domains. Platform management staff can configure the platform and exploit diagnostic data.

The CAREUP platform automatically collects large amounts of data through the IoT network (sensing and controlling domain). However, for some analog devices, information should be entered manually. The CAREUP platform relies on computer-based questionnaires designed by an expert group comprising members of project partner organizations.

CAREUP visualizes changes in health status and compares these with self-defined health goals, generating awareness about the effects of intervention on the user’s health status. The care plan composition module uses monitoring results and guidelines to generate a personalized plan of activities covering IC domains.

### 5.5. Resource and Interchange Domain

This domain comprises modules responsible for the storage, processing, and sharing of data with other domains. The user database and data exchange are handled by PostgreSQL, which is a relational database. The main reasons for that choice are the increased relationship between data entities found during CAREUP platform design and the need for more robust, fast, and secure technology. Although a noSQL database has the advantage of being able to manage massive amounts of data and be easily scalable, at a particular moment, a relational database like PostgreSQL becomes necessary to manage complex relationships in a fast and reliable way while providing a great set of tools to the developers and the maintainers of the system.

The security of data is ensured by implementing a data management plan, which defines several procedures for maintaining data privacy. The data management plan follows the GDPR obligations and imposes the following conditions on data collection and processing:

- The data types collected by the platform are limited to those that are crucial for its operation. The platform does not collect unnecessary data for possible future use.
- The redundancy and duplication of data across databases is avoided to improve security.
- The data sent to the platform is pseudonymized, which makes it more difficult to trace them back to particular users. Each user is assigned a unique, randomly generated identifier that is used in place of her/his personal information.
- The data links between the applications and the platform’s cloud services are encrypted.
- The user’s personal data are stored in a separate, secure database, ensuring they are detached from their medical data.

The data access control module verifies the correctness and validity of users’ logins and passwords (authorization) and provides access to the O&M platform functionalities through login/password authentication. Both functionalities are implemented in the API module, which provides JWT and OAuth 2 authentication mechanisms to secure access and data exchange.

Data preprocessing prepares data that will be processed in the evaluation and prediction module, which performs threemain functions: evaluation of the primary user’s performance in IC domains, calculation of total IC measure, and prediction of IC changes.

### 5.6. Operations and Maintenance Domain

The operation and maintenance (O&M) module comprises access, remote software installation and configuration, remote diagnostics, and system database services.

Remote software installation and configuration enable the deployment of basic functionalities and modules to the core server and portable devices (a tablet) using an automatically configured Git run in Docker containers. For software deployment and updates of gateways, sensors, and other devices with Internet access enabled, a reverse proxy mechanism is used if necessary. Red Hat Ansible is employed to automatize the procedure.

The design choice for the system database was to implement it in the same database as the user database, i.e., the CAREUP core database implemented in PostgreSQL. The system database stores the following data types related to platform operation and management: credentials for all platform users, including O&M staff; pseudonyms of the users to enable the anonymization of the data; and data describing system configuration (e.g., mapping devices used to link particular locations to particular users, device identifiers and addresses, and devices’ configuration parameters).

### 5.7. Data Sources

Monitoring the user’s health state and providing her/him with personalized care plans requires the collection of data concerning both his/her health parameters and emotions. The data processed by the platform come from several sources:

- Medical devices: blood pressure meters, oximeters, digital scales, blood sugar meters, grip strength meters, sleep trackers, step counters (smartwatch), etc.;
- Positive health questionnaires;
- Cognitive games.

**Figure 3:**
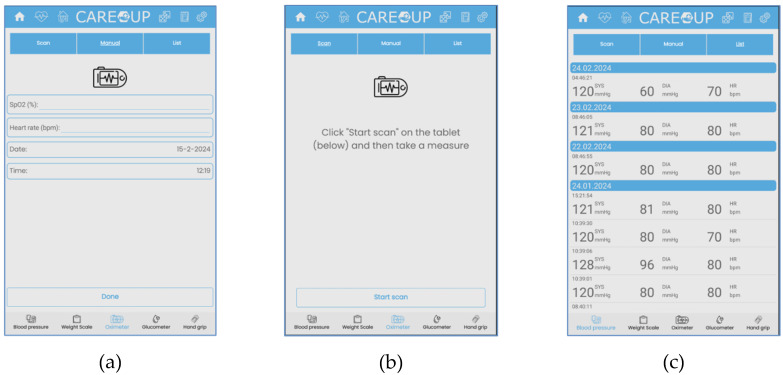
Care Plan application data acquisition options: (**a**) manual input; (**b**) automatic download; (**c**) saved values list presented to the user.

The data from the devices are collected using the Care Plan application. Exemplary screenshots of the application are presented in Figure 3.

The data from the devices may be supplied to the platform in several ways. The first option is addressed to users who use older devices incapable of wireless communication. Such users need to input the results manually, using an intuitive interface. If users possess newer devices equipped with Bluetooth, the data are transferred automatically to the platform. The user can view the recorded values at any time.

**Figure 4:**
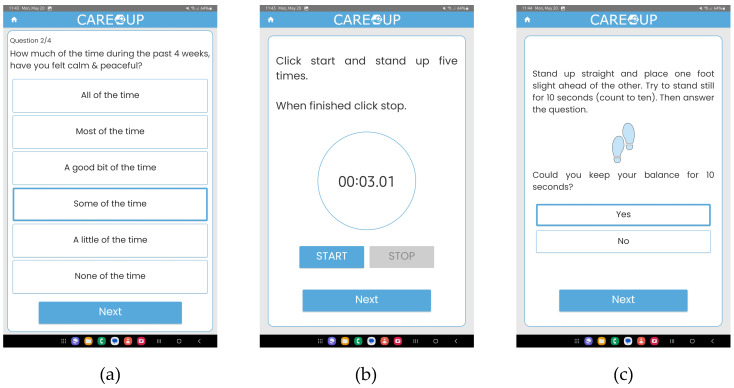
The Positive Health application: (**a**) Positive Health questionnaire; (**b**) locomotion chair stand screen; (**c**) locomotion balance test screen.

The second data source is positive health questionnaires, which allow the platform to assess the user’s state in non-measurable domains such as emotions and self-perceived capabilities. They are implemented in the Positive Health application, exemplary screens of which are presented in Figure 4.

The Positive Health application presents the users with short, three- to four-question questionnaires asking about their current mood and health (Figure 4a). The questions come from the questionnaires used for IC evaluation. Frequent asking of selected questions allows the platform to efficiently update the user’s IC score and present him/her with an actual prediction.

The PH application also implements a short locomotion assessment test based on the Short Physical Performance Battery. The test implements two parts of the SPPB: a chair stand (Figure 4b) and a balance test (Figure 4c). In both of the tests, the user is presented with detailed instructions and is asked to perform a short physical exercise, the completion time of which is measured. The obtained data are used to update the locomotion IC score. As the walking speed test requires the user to take walk of a few meters, it is not possible to implement it using a tablet app. The ability to walk may be assessed based on the smartwatch step-counter output.

**Figure 5:**
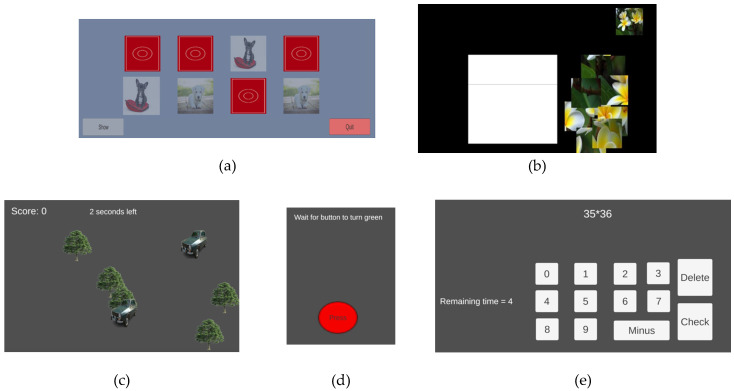
Screenshots cognitive games application: (**a**) match game; (**b**) reaction; (**c**) moving objects; (**d**) puzzle; (**e**) computations.

The last data source is the CAREUP cognitive games. The games have a dual purpose. First, they are used for entertainment and to boost the user’s cognitive abilities [44]. Secondly, changes in achieved scores might be an indicator of a functional decline. The application offers several games, with exemplary screens presented in Figure 5.

In the application, there are five cognitive games available:

- Match Game: The user’s task is to pair cards lying face down to match their face pictures.
- Reaction: The user’s goal is to click on a colored object as soon as it is displayed on the screen;
- Moving objects: The user is asked to click on as many objects of a specified kind as possible in a predefined time;
- Puzzle: The users solve a jig-saw puzzle without receiving indications of the correct/incorrect placement of the pieces;
- Computations: The user performs simple mathematical computations (addition and subtraction) or complex operations (addition, subtraction, multiplication, and division) in a predefined time.

The scores of the games, corresponding to the time needed to complete the puzzle or pair the cards, the reaction time in which the object was clicked, and the number of correctly solved computations or selected elements, are sent to the platform server, where they are stored and may be processed to assess possible functional decline.

## 6. Intrinsic Capacity Evaluation and Prediction

### 6.1. IC Definition

The concept of Intrinsic Capacity (IC) was introduced by the World Health Organization (WHO) as a tool for understanding individuals’ capabilities and functional abilities as they age. It is part of a broader framework called the “World Report on Ageing and Health”, which aims to address the challenges and opportunities of an aging population. Intrinsic capacity refers to the composite of all physical and mental capacities an individual possesses, encompassing their ability to perform various activities and maintain well-being.

The concept of IC is crucial in the context of aging, as it highlights the importance of viewing older adults holistically, recognizing their diverse capabilities, and understanding that age-related changes are not solely determined by chronological age but are influenced by multiple factors. By identifying and supporting intrinsic capacity, it becomes possible to promote healthy aging, enhance functional abilities, and create environments that facilitate active and productive aging.

The WHO recognizes five key IC domains that contribute to an individual’s overall functional ability:

- Locomotion refers to an individual’s ability to move independently, including walking, transferring, and maintaining balance.
- Cognition involves mental processes such as memory, attention, learning, and problem-solving abilities.
- Vitality pertains to an individual’s energy levels, enthusiasm, and general well-being.
- The psychological domain focuses on an individual’s emotional resilience, coping mechanisms, and psychological well-being.
- Sensory capacity encompasses the ability to perceive and interpret information through the senses, such as vision and hearing.

These capacities are influenced by a combination of personal factors, such as physiological and psychological attributes, as well as external factors, like the environment and lifestyle choices.

### 6.2. IC Evaluation

The evaluation of intrinsic capacity is a complex and evolving area of research. Currently, there is no universally recognized standard for evaluating intrinsic capacity, so various assessment tools and methods are used to measure its different domains. These approaches typically depend on widely used questionnaires For example, IC evaluation in the psychological domain may be performed using the Geriatric Depression Scale (GDS-15) or the Patient Health Questionnaire (PHQ-9). In the CAREUP platform, the IC components are scored in the 0–1 range based on the results of the questionnaires filled out by the users and performed measurements.

The locomotion IC is evaluated based on the results of the Short Physical Performance Battery (SPPB) test [45]. The SPPB involves three physical exercises that test the user’s balance, walking speed, and ability to change positions from sitting to standing. The IC in the locomotion domain is computed in the following manner:

(1) $${\mathrm{IC}}_{\mathrm{Locomotion}}=\frac{\mathrm{SPPB}}{\max(\mathrm{SPPB})}$$

where SPPB is the score on the SPPB test.

The vitality IC is a composite value of reported energy levels and grip-strength measurements:

(2) $${\mathrm{IC}}_{\mathrm{Vitality}}=\min\left(0.13\cdot \left(6-\mathrm{SF}12\left[10\right]\right)+0.09\cdot \mathrm{Grip}\text{\_}{\mathrm{strength}}_{\left[\mathrm{kg}\right]},1\right)$$

where SF12[10] is the answer to the energy level-related question from the SF-12 questionnaire and Grip_strength is the measured grip strength. The coefficients in the equation are based on the confirmatory factor analysis performed in [46].

The psychological IC is assessed using the Patient Health Questionnaire (PHQ-9) [47]. The score is computed based on the following widely adopted cut-offs:

(3) $${\mathrm{IC}}_{\mathrm{Psychology}}=\left\{\begin{array}{cc} 1.00, & \mathrm{if}\;0\leq \mathrm{PHQ}\leq 4\\ 0.75, & \mathrm{if}\;5\leq \mathrm{PHQ}\leq 9\\ 0.50, & \mathrm{if}\;10\leq \mathrm{PHQ}\leq 14\\ 0.25, & \mathrm{if}\;15\leq \mathrm{PHQ}\leq 19\\ \frac{27-\mathrm{PHQ}}{32}, & \mathrm{if}\;19<\mathrm{PHQ} \end{array}\right.$$

where PHQ is the total score on the PHQ-9 questionnaire. For high PHQ-9 scores, indicating severe depression, the IC value linearly falls to 0.

Cognitive performance is evaluated based on the Montreal Cognitive Assessment Test (MOCA). The score is the MOCA score scaled to the 0–1 range.

(4) $${\mathrm{IC}}_{\mathrm{Cognition}}=\frac{\mathrm{MOCA}}{\max(\mathrm{MOCA})}$$

The sensory IC is assessed based on self-rated hearing (left and right) and vision (far and near). The IC is a composite of the answers to the above questions:

(5) $${\mathrm{IC}}_{\mathrm{Sensory}}=\frac{H_{\mathrm{left}}+H_{\mathrm{right}}+V_{\mathrm{far}}+V_{\mathrm{near}}}{20}$$

where $H_{\mathrm{left}}$, $H_{\mathrm{right}}$, $V_{\mathrm{far}}$, and $V_{\mathrm{near}}$ are hearing and vision capabilities rated from 1 to 5 (higher scores are better).

The IC values are computed based on the answers to the questionnaires and measurement results recorded with CAREUP. The initial evaluation in the IC domains performed when the user starts using the platform (fills out the questionnaires and performs relevant measurements for the first time).

The IC values are then updated on a daily basis when relevant new data become available. The questionnaire answers essential for IC computation are collected using the Positive Health application. The questions are asked in monthly cycles to not overburden the user with repetitive questionnaires. The questions concerning more rapidly changing aspects, e.g., sleep quality, are asked several times during that period, whereas those related to slowly changing areas like sensory capacity are asked once every four weeks. To assure that the results of measurements with medical devices are up to date, the platform periodically reminds the user to perform them.

### 6.3. IC Prediction

#### 6.3.1. IC Prediction Concept

**Figure 6:**
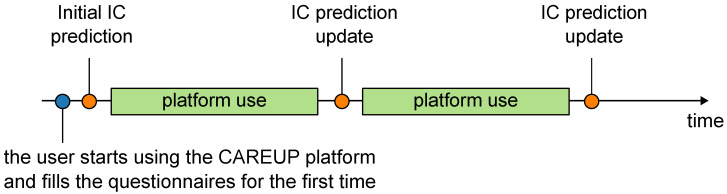
The concept of IC prediction over the CAREUP platform use cycle.

One of the novelties of the CAREUP platform is its ability to supply the user with a prediction of their future performance in the intrinsic capacity domains. IC prediction is performed separately in five domains according to the cycle presented in Figure 6.

Users are supplied with an initial IC prediction alongside their current IC as soon as they start to use the platform. This prediction is based solely on the user’s socio-demographic data and the answers to the first round of questionnaires. The obtained prediction is then updated periodically, considering new data gathered throughout the platform’s use.

**Figure 7:**
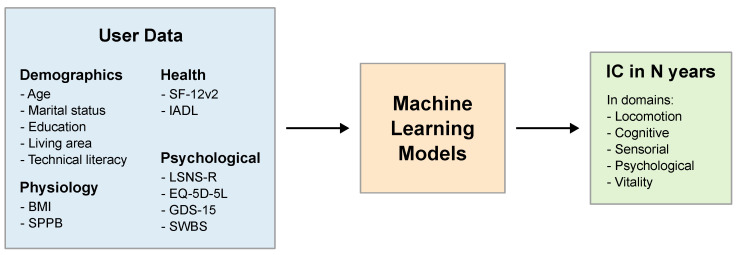
The concept of the IC prediction model.

Predictions of the user’s performance in the IC domains are performed using machine learning models. Figure 7 presents the general idea behind IC prediction.

The prognosis of the user’s future IC is based on his/her socio-demographic information, physiology, current health, and psychological status. The results are discrete—each feature is a single value, e.g., measured BMI or singular answers to selected questions from the questionnaires. The data are passed to a machine learning model, which processes them and outputs the proposed IC over a horizon of several (e.g., two) years. IC prognosis is repeated every week based on the updated incoming data from the platform.

#### 6.3.2. Input Data

At the moment, the amount of data collected with the CAREUP platform is not large enough to effectively train machine learning models. Therefore, the models were trained on publicly available datasets of the English Longitudinal Study of Ageing (ELSA UK) [48]. In the project, we used a harmonized version of the ELSA UK dataset, which contains data gathered in the United Kingdom in nine waves from 2002 to 2019. It includes data from almost twenty thousand individuals who took part in at least one study wave. The data gathered in each wave comprise information about various aspects of the participant’s life, including demographics; health; family; finances; employment; and performance in several areas, such as cognition and physical capacity. The waves were conducted in two-year periods.

The datasets used to train the IC prediction models were constructed by taking the person’s answers from one wave and combining them with a and IC score label computed based on answers from the next consecutive wave. As mentioned above, the ELSA UK waves were separated by two years. However, the physiological measurements needed for locomotion and vitality IC assessment performed made every four years due to that fact those ICs can be predicted over a four-year horizon rather than over two years, as in the case of the other domains. The list of the variables used by the models is presented in Table 1.

As the scope of ELSA UK does not cover all of the data gathered by the CAREUP platform, the models use only selected CAREUP parameters, which have their counterparts in the ELSA UK dataset. In the future, when more CAREUP-specific data become available, the variable selection process will be repeated. As shown in [49], not all variables are relevant to IC prognosis in each of the domains.

All models use a similar set of variables. The only exceptions are grip strength, which is used solely by the vitality model, and SPPB results (balance, walking speed, and chair stands), which are used by the locomotion model. The omission of these values in other models results from the fact that for these variables, the number of available results in the training dataset was low. This was caused by the fact that, in the ELSA UK study, both SPPB and grip-strength measurements had to be performed by qualified personnel; thus, those answers are missing for many participants. The number of examples in each dataset varied due to the differing availability of the future IC scores (the labels were not imputed). The IC scores were calculated using the rules described in Section 6.2.

Before being passed to the models, the variables are preprocessed. The answers to questions are scaled to the 0–1 range, and the categorical questions, for which the answers are not quantitative, are hot-encoded (in CAREUP, only concerning marital status). The scores of MOCA and PHQ9 are additionally standardized to resolve the problem of differing scales used for depression and cognitive capacity evaluation in CAREUP and ELSA (custom cognitive questions and the Center for Epidemiologic Studies Depression Scale (CESD), respectively). The means and standard deviations for ELSA UK tests were calculated based on the available data. The corresponding values for MOCA and PHQ-9 were taken from the literature. For MOCA, based on [50], the mean score was 23.25, with a standard deviation of 4.82. For PHQ-9, based on [47,51,52,53], the mean was 5.79, with a standard deviation of 5.8.

#### 6.3.3. Prediction Models

During the project, we considered using different types of machine learning regression methods for IC score prognosis. The tested methods include:

- Linear regression;
- Random forest regression, tested for different numbers of trees (10, 50, 100, 200, 300, and 500);
- XgBoost, tested for different numbers of trees (10, 50, 100, 200, 300, and 500);
- Dense neural networks (DNNs) with different numbers of layers and neurons (Table 2).

The models were implemented using Python. Linear regression and random forest used their standard implementations from the scikit-learn [54] and DMLC XgBoost libraries [55]. The neural networks were implemented in Pytorch and consisted of several dense layers, including a varying number of neurons. Each dense layer was followed by batch normalization to improve the models’ generalization capabilities. The output layer always included one neuron with a linear activation function. We tested several combinations of layers and numbers of neurons and selected the most accurate based on MSE and MAE. The details of the architectures are presented in Table 2.

The accuracies of the proposed methods were tested on the prepared ELSA UK datasets. Each of the architectures was evaluated using k-fold cross-validation with k equal to 10. In this validation scheme, the dataset is shuffled and partitioned into training and test datasets. The partition is repeated ten times, taking a different portion of data as a test dataset each time. The model is then trained and tested on the obtained data splits, which ensures that the model’s performance was tested for all observations. The resulting accuracy is the average of metrics computed for the ten splits. The methods’accuracies were assessed based on four metrics:

- Mean Squared Error (MSE);
- Mean Absolute Error (MAE);
- Coefficient of determination (R2);
- Median Absolute Error (MedAE).

The data supplied to the above methods were preprocessed according to the same scheme as described in Section 6.3.2. The obtained accuracy metrics are presented in Table 3. The table includes the best results achieved for each of the methods. The MAE did not exceed 0.14 for any of the domains, which is an acceptable result, given the complexity of the problem.

The best performance was achieved for the locomotion domain, in which the mean absolute error was at a level of 0.0666. The prediction errors in this domain may result from how the SPPB test components are scored. Even a small change in walking speed may cause the measured time to fall between different thresholds, which would cause a significant change in the total score.

A slightly lower accuracy was obtained for vitality and cognition prediction. The lower quality of vitality prognosis might result from the relatively small number of available grip-strength measurements. In the case of cognition, the accuracy might have been higher if the dataset included additional typically used data as processed parameters of MRI scans. However, such data cannot be obtained using everyday ICT platforms.

The MAE for sensory IC prediction was about 0.09. Predicting sensory performance is especially hard, as the questions concern subjective ratings of sight and hearing (even when using glasses or hearing aids) and not the actual health state of the person. It was observed that for some patients, even after observing a falling trend in visual capacity over several waves, there was a sudden increase due to, e.g., having cataracts removed.

The worst accuracy was achieved for the psychological domain. Predicting whether a person is at risk of depression or lowered mood is a challenging task due to the presence of random contributing factors independent of the person’s health state, e.g., the death of a loved one or job loss.

For all IC domains, the results obtained using the tree-based methods and neural networks had a similar accuracy. This suggests that for this prediction problem (variables and the predicted values), the accuracies may be at their theoretical limits.

Given the minor differences in the methods’ accuracies, we decided to implement the prediction algorithms as neural networks, as they have some advantages over the other methods. First of all, in the future, when the system gathers enough time series data from sensors and questionnaires, the models will be updated to accept more types of data. When it comes to unstructured data such as time series, neural networks have a significant advantage [56] over tree-based methods. They allow for efficient encoding of different types of data, as they do not require hand engineering of the input features (the feature extractor is trained on data). By implementing NN-based models now, NN-enabled infrastructure will be already in place when a new batch of models is trained and ready to be deployed.

Additionally, historically, NNs have shown promising performance improvement when training datasets are enlarged. Moreover, NNs support online training on incoming new data samples, which is welcomed, as the datasets used for IC prediction will gradually grow with platform use.

## 7. Pilots and Exemplary Results

The fully developed platform was tested during the pilots, which were conducted by the end-user partners (EURAG, INRCA, and AAIF) in their respective countries (Italy, Austria, and Romania). The countries selected for pilots differed in terms of e-literacy of the older population. According to a report by the European Union Agency for Fundamental Rights [57], for those countries, the highest percentage of older people with basic digital skills was observed in Austria. Both Italy and Romania are below the EU average, with Romania ranking second to last in the whole EU.

During the pilot, 64 older adults used the CAREUP system in their living environments. The objectives of the final phase were to assess the primary outcomes (adherence, usability, and acceptance) and the secondary outcomes (physical health, perceived loneliness, social interaction, quality of life, and cognitive health). The platform was also tested by 15 caregivers.

The tested platform setup comprised the complete platform, including applications and several sensors. A list of the devices that were supplied to the users is presented in Table 4.

**Figure 8:**
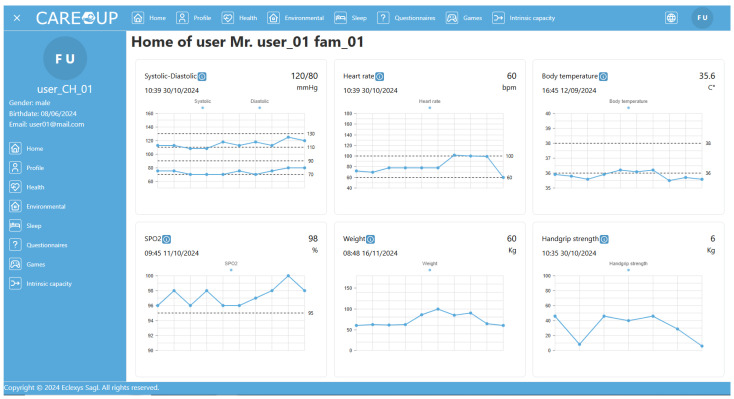
Presentation of medical measurement results (dashboard).

**Figure 9:**
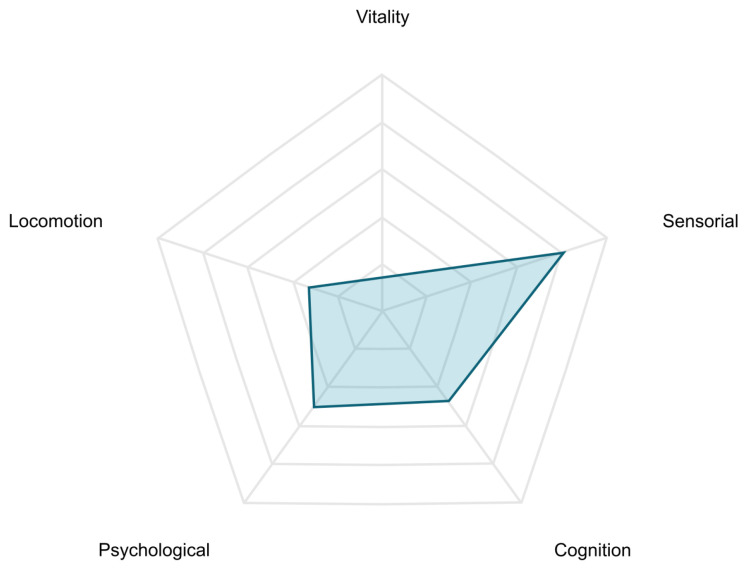
Presentation of current intrinsic capacity values.

**Figure 10:**
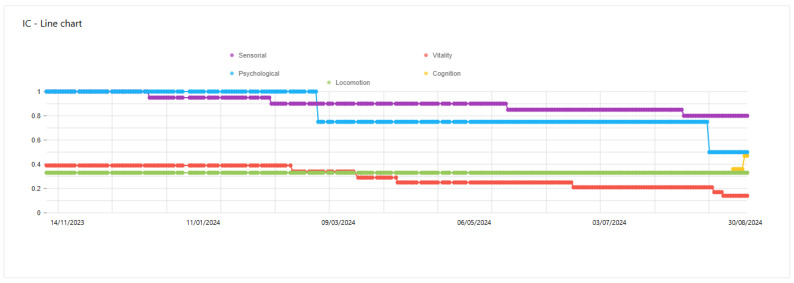
Presentation of intrinsic capacity changes over time.

The presented list of devices covers only the models that were offered to the users by the end-user partners. If the users already owned analogous devices and did not want to use another, they used the manual measurement input of the Care Plan application to save their health parameters to the platform. Exemplary results achieved for the users are presented in Figure 8, Figure 9 and Figure 10.

The medical measurement results (blood pressure, saturation, temperature, and weight) are presented as time series on graphs organized in a single dashboard. The value of the most recent result is also in a numerical form.

The current value of the intrinsic capacity is presented in the form of an intuitive radar graph. The graph shows performance in all five domains. As there is no standardized formula for overall amount of intrinsic capacity available, we decided not to present it directly. The area surrounded by the curve on the radar graph might be treated as an indicator of overall performance instead.

The changes in intrinsic capacity in the domains are presented in time-series graphs. The observed changes may provide caregivers or doctors with insight as to how the older adult’s performance changes and how to take appropriate action to delay the decline.

In the pilots, we also analyzed the intensity of active use of the platformed by both the primary users and their caregivers. The analysis was performed based on login history and timestamps of questionnaire answers transmitted to the platform. A basic comparison of platform use statistics for primary users is presented in Table 5.

For Austria and Italy, the user participation was at a similar level despite significant differences in the countries’ digital literacy rates. Moreover, in Italy, there were several users who actively used the platform practically every day, whereas the most active user in Austria logged in every second day. In Romania, the platform was used less frequently. In all of the countries, 75% of the users used the platform more than one or two times per week. This number conforms to ICT platform acceptance rates reported in other studies [58,59], ranging from 70 to 90%. Despite the lower use intensity recorded for some users, the time granularity of the results should still allow for effective IC change monitoring.

Compared to the other available studies, the intensity of the platform use was at a satisfactory level. Findings for the InnoWell well-being platform [60] deployed in Australia have shown that it is tough to maintain the user’s involvement. In the described study, 84% of participants completed the 90-day trial, out of which only half logged in more than once. None of the users used the platform more than ten times. Better participation was recorded in the training program described in [61]. In the program, the users were asked to play serious games, aiming to improve their cognitive capabilities. Even though some users were aware of potential gains, only half met the minimal adherence goal of 10 min of daily game play.

Caregivers used the platform in a similar fashion in all of the piloting countries. Caregivers usually accessed the CAREUP portal once or twice a week to monitor the progress of the primary users they take care of.

## 8. Conclusions

This paper describes CAREUP, an integrated care platform supporting older adults based on intrinsic capacity monitoring. The platform, besides typical functionalities of storing medical measurements and providing personalized recommendations, provides novel functionalities of intrinsic capacity evaluation and machine learning-based prediction. Prediction is performed over a two-year horizon (four years in the case of locomotion and vitality capacities). The obtained accuracy is acceptable, given the horizon and the lack of specialized measurements (MRIs or genes).

The developed platform’s operation was positively tested during pilots performed with the participation of older adults and their caregivers in three countries with differing levels of digital fluency: Austria, Italy, and Romania. Besides standard support and updates, the platform will be developed further. As more data from users arrive, the IC prediction models will be updated to include more data that are not covered by openly available datasets.

## Figures and Tables

**Table 1:** The variables used by the specific models.

Data Source	Variables	Locomotion	Sensory	Psychology	Cognition	Vitality
Socio-demographics	Age, sex, marital status (one-hot-encoded), and education	x	x	x	x	x
ine Physiology	BMI	x	x	x	x	x
ine Sensorial performance	Left/right hearing and near/far vision	x	x	x	x	x
ine Locomotion parameters	SPPB results (balance, walking, and chair stands)	x				
ine Cognitive performance	Result of MOCA	x	x	x	x	x
ine Depression evaluation	result of PHQ-9	x	x	x	x	x
ine Health Status SF-12 questionnaire	Answers to questions 1, 2, 3, 4, 5, 6, 8, 10, and 11	x	x	x	x	x
ine Instrumental Activities of Daily Life questionnaire	Answers to questions 1, 2, 3, 4, 5, 7, and 8	x	x	x	x	x
ine UCLA Loneliness Scale questionnaire	Answers to questions: 1, 2, 4, 11, and 14	x	x	x	x	x
ine Lubben Social Network Scale questionnaire	Answers to questions 6 and 12	x	x	x	x	x
ine Health Questionnaire Eq-5D-5L	Answers to questions 1, 2, 4, and 5	x	x	x	x	x
ine Other measurements	Grip strength					x
ine DATASET SIZE		3890	14,312	14,204	14,245	7720

**Table 2:** Architecture of the IC prediction neural networks models.

Domain	Input Size	Layers	Output
Locomotion	48	Four layers with 32, 32, 32, 32 neurons	Locomotion IC in 0–1 range
Sensory	43	Four layers with 16, 16, 16, 16 neurons	Sensory IC in 0–1 range
Psychology	43	Four layers with 16, 16, 16, 16 neurons	Psychology IC in 0–1 range
Cognition	43	Three layers with 16, 32, 16 neurons	Cognition IC in 0–1 range
Vitality	44	Four layers with 8, 16, 16, 8 neurons	Vitality IC in 0–1 range

**Table 3:** Performance metrics of the tested IC prediction models.

Domain	Method	Test Data				Train Data			
		MSE	MAE	R2	MedAE	MSE	MAE	R2	MedAE
Locomotion	Linear Regression	0.0080	0.0675	0.3790	0.0520	0.0077	0.0664	0.4064	0.0515
	Random Forest (trees = 200)	0.0083	0.0695	0.3603	0.0536	0.0011	0.0257	0.9127	0.0196
	XgBoost (estimators = 10)	0.0085	0.0700	0.3404	0.0501	0.0050	0.0552	0.6158	0.0430
	Neural Network	0.0085	0.0666	0.3878	0.0478	0.0058	0.0550	0.5555	0.0398
Vitality	Linear Regression	0.0088	0.0729	0.5817	0.0594	0.0087	0.0725	0.5877	0.0588
	Random Forest (trees = 300)	0.0093	0.0751	0.5583	0.0616	0.0013	0.0276	0.9399	0.0226
	XgBoost (estimators = 10)	0.0092	0.0747	0.5645	0.0616	0.007	0.0656	0.6691	0.0543
	Neural Network	0.0085	0.0727	0.5818	0.0617	0.0085	0.0724	0.5962	0.0596
Psychology	Linear Regression	0.0320	0.1255	0.3373	0.0854	0.0317	0.1250	0.3429	0.0849
	Random Forest (trees = 300)	0.0334	0.1310	0.3082	0.0903	0.0046	0.0483	0.9055	0.0330
	XgBoost (estimators = 10)	0.0328	0.1277	0.3206	0.0827	0.0263	0.1162	0.4555	0.0787
	Neural Network	0.0380	0.1342	0.2642	0.0838	0.0313	0.1246	0.3490	0.0822
Cognition	Linear Regression	0.0130	0.0887	0.4161	0.0723	0.0129	0.0884	0.4210	0.0720
	Random Forest (trees = 300)	0.0136	0.0909	0.3919	0.0743	0.0019	0.0335	0.9169	0.0272
	XgBoost (estimators = 10)	0.0132	0.0895	0.4068	0.0722	0.0114	0.0834	0.4913	0.0680
	Neural Network	0.0127	0.0875	0.4179	0.0708	0.0127	0.0877	0.4303	0.0720
Sensory	Linear Regression	0.0136	0.0931	0.4277	0.0778	0.0135	0.0928	0.4318	0.0777
	Random Forest (trees = 300)	0.0141	0.0948	0.4068	0.0795	0.0019	0.0350	0.9189	0.0292
	XgBoost (estimators = 10)	0.0138	0.0940	0.4182	0.0795	0.0121	0.0880	0.4927	0.0746
	Neural Network	0.0136	0.0938	0.3905	0.0814	0.0135	0.0921	0.4343	0.0779

**Table 4:** List of devices used during the pilot studies.

Device	Model	Measured Parameters
Weight scale	iHealth HS2S FIT	Weight and body composition
Blood pressure meter	iHealth Track KN-550BT	Blood pressure
Oximeter	iHealth PO3M	Blood saturation and pulse
Hand grip-strength meter	GRIPX Digital Hand Dynamometer	Hand grip strength
Sleep tracker	Withings Sleep Tracking Mat WSM02	Sleep duration
Thermometer	Withings SCT01	Body temperature
Smartwatch	Starmax S50	Step count
Tablet	Lenovo Tab M10 or a tablet owned by the user	Questionnaires

**Table 5:** Average time between active platform use in days.

Country	Average	Min	25th Perc.	Median	75th Perc.	Max
Austria	3.48	2.00	2.49	3.20	3.40	8.33
Italy	3.47	1.07	2.05	2.92	3.95	9.71
Romania	4.67	1.97	3.46	4.33	4.80	11.00

## Data Availability

The datasets presented in this article are not readily available because they include health data currently being processed. Requests to access the datasets should be directed to Jaouhar Ayadi of Eclexys at jaouhar.ayadi@eclexys.com.
